# U-Behavior: a method to promote spaced and interleaved retrieval practice in challenging undergraduate microbiology and biomedical sciences courses

**DOI:** 10.1099/acmi.0.001226.v3

**Published:** 2026-08-04

**Authors:** James E. Folkestad, Hannah Hausman, Marcia Moraes, Katriana Popichak, Jennifer McLean, Erica Suchman, Thomas Rausch

**Affiliations:** 1 School of Education, Colorado State University, Fort Collins, USA; 2 Psychology, University of California, Santa Cruz, Santa Cruz, USA; 3 Computer Science, Colorado State University, Fort Collins, USA; 4 Microbiology, Immunology, and Pathology, Colorado State University, Fort Collins, USA; 5 Colorado State University, Fort Collins, USA

**Keywords:** behaviour change, coaching study strategies, interleaving, learning management system, microbiology education, retrieval practice, spaced practice, undergraduate teaching

## Abstract

Students in demanding microbiology and biomedical sciences courses often rely on suboptimal study strategies, such as massed practice, despite evidence favouring spaced retrieval and interleaving for durable learning, where durable learning is learning that is retained across longer periods of time. We developed U-Behavior, a learning management system-integrated pedagogy tool that coaches these behaviours through instruction, personalized visualizations of quiz attempt patterns [retrieval practice activity (RPA) graphs], reflections and behavioural feedback. In two experiments with identical RPAs in gateway (VMBS 100, introductory biomedical sciences) and upper division (MIP 300, general microbiology), we compared U-Behavior to low-stakes quizzing (control). U-Behavior significantly increased spacing and interleaving (large effects, ds >1.75) without raising total quiz attempts. Approximately half of U-Behavior students achieved optimal practice levels. No significant between-condition differences appeared in midterm/final exams, likely due to partial adoption. However, higher spacing scores predicted better exam performance (small-to-medium effects) beyond prior Grade Point Average (GPA) and attempt quantity. U-Behavior provides a scalable, behaviour-focused approach to enhance study quality in rigorous microbiology education.

## Data summary

The underlying data for this study consist of anonymised, processed student-level records from two quasi-/randomized controlled experiments evaluating the U-Behavior teaching and learning method in undergraduate Science, Technology, Engineering, and Mathematics (STEM) courses. These datasets include retrieval practice activity (RPA) usage logs (timestamps of quiz attempts, scores, derived spacing and mixing behaviour scores), U-Behavior (UB)-rubric levels, total RPA attempts, exam performance (midterm/final exams), overall course grades and prior academic performance covariates [high school Grade Point Average (GPA) and/or university GPA]. All data were extracted from the university’s learning management system (LMS) and course records and then de-identified to comply with institutional review board approvals, FERPA regulations and participant consent.

Raw individual timestamps and identifiable information are not shared. Instead, the deposited datasets provide the processed, aggregated and summarized variables used for all statistical analyses reported in the manuscript (e.g. spacing scores, mixing scores, UB-rubric levels, total attempts, exam scores, regressions, descriptive statistics and effect sizes). These allow independent verification of the key findings – significant increases in spacing and interleaving under U-Behavior, no overall condition differences in exam performance and predictive relationships between spacing quality and learning outcomes – and replication of the reported regressions and group comparisons.

The datasets have been deposited in Dryad, in accordance with Access Microbiology’s Open Data policy.

- Repository: Dryad (data has been uploaded but holding on publication for review)
- DOI: 10.5061/dryad.cnp5hqck8
- URL: https://doi.org/10.5061/dryad.cnp5hqck8

The deposit includes two main Excel files:

1. VMBS100 - Dryad.xlsx – Processed data from Experiment 2 (Introduction to Biomedical Sciences, VMBS 100; randomized assignment; freshman-level gateway course). It contains variables such as Consent, Condition, Attention Value, Midterm, Final Exam, Final Score, Final Grade, Retrieval Practice Activities, Spacing (%), Mixing (%), Behaviour Check, UB-Level (1–4), Original-UB-Grade, HS_GPA, AttemptsRPA11, AttemptsRPA12 and Total Attempts.
2. MIP300 001 - Dryad.xlsx – Processed data from Experiment 3 (General Microbiology, MIP 300; quasi-experimental by section; upper-division foundational course). It contains variables such as Section, Consent, Condition, AttentionValue, Exam 1, Exam 2, Exam 3, Final Exam, ExamsFinalScore, Final Exam Grade, Final Score, Final Grade, Retrieval Practice Activities, Spacing (%), Mixing (%), UB-Level (1–4), Original-UB-Grade, U-Behavior Final Points, Practice Behaviour Check ALL, U-Behavior Final Score, Total Attempts, HS_GPA and CSU_GPA.

No custom software, code packages or proprietary tools were generated beyond standard LMS quiz functionality and spreadsheet processing. Any scripts used for deriving spacing/mixing scores or running regressions (e.g. in R or Python) are not deposited but can be requested from the corresponding author subject to institutional approval. Full raw LMS logs are unavailable for public deposit due to privacy constraints.

## Introduction

Durable learning, the retention of knowledge in a form that supports future application, beyond performance on a single examination, depends on a small set of well-established study behaviours. Among these, spaced practice (distributing study sessions across multiple days rather than concentrating them immediately before an exam), retrieval practice (actively recalling information from memory rather than rereading it) and interleaving (alternating among related topics within a study session, rather than studying one topic to completion before moving on) consistently outperform massed practice (back-to-back study of the same content, colloquially ‘cramming’) in laboratory and classroom settings [1–4]. The effects are largest on long-delay, content-rich assessments – exactly the conditions encountered in upper-level science courses, where new material must be retained across weeks of additional instruction and integrated with content covered later in the semester.

Despite robust evidence for these strategies, university students rarely adopt them spontaneously, even when they have been told that the strategies work. Surveys and observational studies consistently report that students default to massed rereading and recognition-based review near exams [5–7]. This pattern reflects what social and behavioural scientists call the intention-behaviour gap [8]: a robust, replicated dissociation between what people intend to do and what they actually do, particularly for effortful behaviours with delayed rewards. Direct instruction in effective study strategies – telling students that spacing and retrieval work – produces, at best, short-lived self-reported change and rarely shifts measurable practice behaviour in higher-education settings [9].

Closing this gap requires moving from instruction to behaviour change. Hattie [10] argues that the strongest leverage point in higher education is visible learning: making the learning process observable to students, so that they can compare their current behaviour against effective patterns and act on the discrepancy. Behaviour-change frameworks complement this principle by specifying that durable change typically requires self-monitoring, feedback, goal setting and a low-stakes incentive structure rather than information alone [11]. The pedagogical question is therefore not whether students should space and interleave their practice – the evidence is clear – but how to design course-embedded scaffolds that move students from intending to do so to actually doing so.

*U-Behavior* is a pedagogical approach that wraps standard low-stakes learning management system (LMS) quizzes which we call retrieval practice activities (RPAs) with three behaviour-change components – a personalized RPA graph, periodic reflection assignments and a low-stakes rubric grade – designed to close the intention-behaviour gap by making practice patterns visible and rewarding spaced and interleaved practice [12].

We tested U-Behavior in two undergraduate microbiology and biomedical sciences courses at a large US public university: VMBS 100 (Introduction to Biomedical Sciences), a gateway course – an introductory course required for entry into a degree program – taken predominantly by first-year students enrolling as biomedical sciences majors (students who have declared biomedical sciences as their primary undergraduate subject specialization), and MIP 300 (General Microbiology), an upper-division course taken predominantly by third-year microbiology majors and equivalents (upper division refers to coursework in the latter half of a typical 4-year US undergraduate degree, requiring prerequisite preparation). The two courses differ markedly in content density and required retention depth, providing a natural test of whether U-Behavior’s effect on practice behaviour and learning outcomes generalizes across course difficulty.

We ask three questions. First, does U-Behavior increase spaced and interleaved practice relative to a strong-control low-stakes quizzing condition that delivers the same RPAs and the same basic instructions? Second, among students in the U-Behavior condition, does the quality of practice behaviour – captured as a four-level rubric score derived from RPA timestamps – predict performance on the midterm (a mid-semester comprehensive exam covering the first half of the course’s content) and final exam (an end-of-semester cumulative exam covering all content) in each course? And third, does the relationship between practice quality and exam performance differ between the introductory and upper-division courses, in a way that is consistent with the durable-learning rationale for the intervention?

## Methods

The following outlines the methods used in this study.

### Setting

Research occurred at a large Western US land-grant university (~24,000 undergraduates; diverse demographics in STEM-heavy courses).

### Conditions

#### What is U-Behavior?

U-Behavior is a behaviour-change wrapper around standard low-stakes LMS quizzing. The underlying quizzes or RPAs are identical to those used in the control condition. U-Behavior adds four components on top of those RPAs: (a) a brief tutorial on the cognitive science of spacing and interleaving; (b) a personalized RPA graph, a visible-form learning analytic that displays each student’s own practice timeline; (c) periodic reflection assignments that ask students to compare their RPA graph to prototype patterns and consider behavioural change; and (d) a low-stakes rubric grade applied to the final RPA graph that rewards spaced and interleaved practice. U-Behavior is therefore a visible-form learning analytic and reflection, plus rubric layered onto the same quizzing affordance the control group receives.

**Fig. 1.**
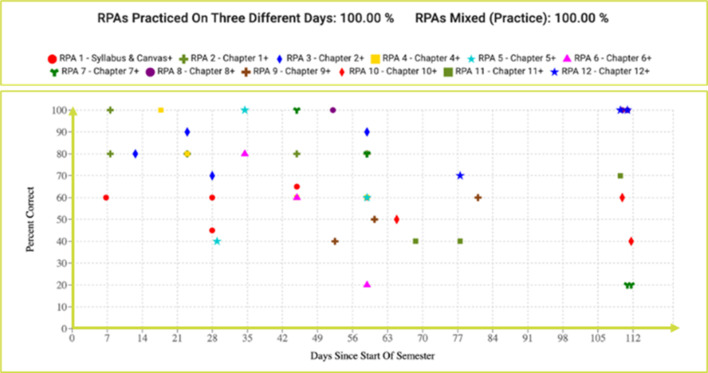
Student’s RPA graph. *Note*: Example RPA graph indicating each RPA attempt by a student since the start of the semester (X-axis) against the percentage correct for that attempt (Y-axis). Students can generate their RPA graph at any time during the semester using the U-Behavior LMS tool.

The U-Behavior condition added four components: (1) a 10-min self-paced tutorial covering the cognitive science of spaced retrieval, interleaving and the use of the RPA graph; (2) the RPA graph, a personalized visualization that each student could open on demand to see their own practice attempts plotted on a timeline (Fig. 1); (3) three reflection assignments distributed at weeks 4, 8 and 12 of the semester, in which students reviewed their current RPA graph, compared it to prototypical high-, medium- and low-practice patterns and considered behavioural change (full details in the Reflection assignments subsection below); and (4) a rubric-based grade applied to the student’s final RPA graph at the end of the semester, with thresholds of 70% Spacing and 40% Mixing for full points (see Measures). The U-Behavior practice component, including reflections, contributed ~5% of the overall course grade, a deliberately low-stakes weighting.

#### What control condition is

Students accessed online RPAs, which were formative, low-stakes quizzes designed for practice rather than assessment. Each RPA was an automatically generated quiz drawing a fresh set of items from a large topic-specific question pool, so no two attempts presented an identical set of questions. RPAs were released weekly, after the corresponding content was covered in class, and remained open for the remainder of the semester. Students could attempt each RPA an unlimited number of times; the highest score earned across attempts contributed to the practice-component points (described below). There was no time limit on individual attempts, and completion was not monitored or required. Practice patterns—including when to attempt which RPAs, how to distribute attempts across days, and whether to alternate between RPAs within a session—were entirely student-driven. The LMS recorded timestamps but did not prompt, schedule, or constrain student behavior. Instructions distributed at the start of the semester briefly explained the benefits of spaced retrieval and interleaved practice and encouraged students to attempt each RPA repeatedly across time, but no further prompts, reminders or visualizations were provided to the control group during the semester. For example, a student who attempted a 10-point quiz five times, earning 3, 5, 8, 9 and 6 would receive a final score of 9 out of 10 for that quiz. The practice-component points from quizzes in the control condition contributed ~5% of the total course grade, the same proportion as the combined quiz and reflection component in the U-Behavior condition, ensuring that overall grade stakes were held constant across conditions. Students in the control condition received identical RPAs to the control group, with the same weekly release schedule, unlimited attempts, no time limit and no completion monitoring.

#### Design rationale: a strong control test of behaviour change

The control condition was constructed to be a deliberately demanding comparison rather than a passive baseline. In both conditions, students had access to the same RPAs, drawn from the same question pools, released on the same weekly schedule, with the same scoring rules; in both conditions, students were given instructions at the start of the semester that explained the benefits of spaced and interleaved practice and encouraged them to attempt each RPA repeatedly across time. The only difference between conditions was the behaviour-change wrapper: the U-Behavior group received the visible-form learning analytic (the RPA graph), the periodic reflection assignments and the rubric grade on the final graph. This isolates the behaviour-change wrapper itself as the active ingredient of the intervention and provides what we consider a strong test of the underlying question: when students are given the affordance for spaced and interleaved practice, including a visual-form learning analytic, do they actually do so? The control condition tests the null hypothesis that instructions alone are sufficient; the U-Behavior condition tests whether layering visible-form feedback and reflection on top of those same instructions produces measurable behavioural change.

#### Reflection assignments

The three reflection assignments occurred at approximately weeks 4, 8 and 12 of the semesters. Each reflection took fewer than 5 min to complete and was delivered through the Canvas LMS as a fully automated module. Each reflection consisted of three steps. First, the student opened the U-Behavior LMS tool and downloaded their current RPA graph as an image file and then uploaded that image as an attachment in Canvas. This ensured that students checked and reflected on their learning behaviour at least three times during the semester. Second, the student was shown three prototype RPA graphs – drawn from anonymised data of past students who exemplified high, medium and low practice patterns at the corresponding point in the semester – and asked to self-identify which prototype most closely matched their own graph. Based on the student’s self-selection, Canvas provided automated feedback: students who self-identified as high-practice received affirming feedback; students who self-identified as medium- or low-practice received coaching feedback that named the specific behaviours associated with higher rubric levels (e.g. distributing practice across more days and returning to a given RPA after attempting others in between) and pointed the student to the resources needed to change those behaviours. Third, the student answered a single open-response prompt: ‘After reviewing the feedback on your RPA practice behaviour, do you plan to change how you use the RPAs in the upcoming weeks (please describe why and how)?’ Instructors provided minimal additional coaching beyond what the automated Canvas module delivered; the ‘coach’ in U-Behavior is primarily the automated comparison system that delivers feedback based on the student’s self-selected prototype match, rather than an individualized human consultation.

### Measures

#### Raw input data

For each student (regardless of condition), we extracted from the LMS the timestamp of every RPA attempt during the semester, along with the RPA identifier (which of the course’s RPAs the attempt was on) and the score earned on that attempt. VMBS 100 included 12 RPAs across the semester; MIP 300 included 10 RPAs. The two practice-behaviour measures defined below were derived from the timestamp+RPA-identifier sequence; the score earned on each attempt was not used in computing practice behaviour but only used for graphing (Y-axis)

#### Behaviour Score 1: Spacing (0–100)

The Spacing score quantifies the proportion of available RPAs that a student practised on three or more distinct calendar days. For each RPA, we counted the number of distinct days on which the student made at least one attempt at that RPA. An RPA was counted toward Spacing if and only if the student attempted it on three or more different days. The Spacing score is then: Spacing %=(number of RPAs practised on ≥3 distinct days)/(total RPAs in the course)×100. A student in VMBS 100 who practised 9 of the 12 RPAs on three or more different days each would have a Spacing score of 9/12=75.0%. The score thus measures how broadly distributed practice was across the RPA set, not the total number of attempts.

#### Behaviour Score 2: Mixing (0–100)

The Mixing score quantifies the proportion of available RPAs for which the student exhibited at least one mixing event. A mixing event for a given RPA occurs when the student returns to that RPA after attempting at least one other RPA in the intervening period. Only one mixing event is required for an RPA to count toward the Mixing score: Mixing =(number of RPAs with ≥1 mixing event)/(total RPAs in the course)×100. In our experience across multiple cohorts and disciplines, Mixing and Spacing are highly correlated, because students who distribute practice across many days nearly always interleave RPAs as a side effect. Across all U-Behavior implementations to date, we have observed only a single student who scored high on Spacing but low on Mixing. We use the terms mixing and interleaving interchangeably; interleaving is used in the cognitive-science literature [1, 2], and mixing is the term used in the U-Behavior interface and rubric, as the term mixing is easier for students to understand.

#### UB-rubric: hierarchical 1–4 mapping

The continuous Spacing and Mixing scores were translated into a hierarchical four-level UB-rubric, originally specified in Moraes *et al*. [12]. Spacing alone determines whether a student qualifies for the upper tiers (Levels 3 and 4); only when Spacing crosses the Level 3+ threshold does Mixing differentiate Level 4 from Level 3 (see Table 1).

**Table 1.** Hierarchical UB-rubric mapping from spacing and mixing scores to levels

Level	Spacing (Score 1)	Mixing (Score 2)	Label
4	≥70%	≥40%	Highly effective practice
3	≥70%	<40%	Effective practice
2	40–69%	Not considered	Less desirable practice
1	<40%	Not considered	Low practice

The 70%/40% thresholds were established in earlier U-Behavior implementations [12] as the boundaries that distinguish students whose practice patterns approximate the spacing-and-interleaving design recommended by the cognitive-science literature on durable learning [1–3] from those whose practice resembles massed study. Note that Level 3 is preserved in the rubric for cases where high-spacing-but-low-mixing occurs, but in the present experiments, no students were classified at Level 3, consistent with the empirical observation that spacing and mixing are highly correlated.

#### Confound free outcomes for within-condition analyses

Because U-Behavior students were awarded points for reaching higher UB-rubric levels (the U-Behavior practice component of the course grade), within-condition analyses by UB-rubric level use only outcomes that are independent of the U-Behavior practice grade: Midterm and Final Exam in VMBS 100 and Exam 1, Exam 2, Exam 3, Final Exam and the four-exam composite (ExamsFinalScore) in MIP 300. FinalScore (which incorporates the practice grade) is not used as a primary outcome in within-UB analyses.

#### Statistical analysis

Between-condition comparisons used Welch’s t-tests and chi-square tests; within-UB-condition comparisons across UB-rubric levels used one-way Welch’s ANOVA (heteroscedasticity-corrected, because Levene’s tests indicated unequal variances across UB-rubric levels) with Games–Howell pairwise contrasts; linear regressions predicted exam performance from Spacing, controlling for prior academic performance High School (HS) GPA in VMBS 100; Colorado State University (CSU) GPA in MIP 300) and total RPA attempts. All analyses were conducted in JASP (JASP Team, 2025; version 0.95.4) [13].

### Experiments

Experiment 1 (randomized assignment): VMBS 100 (freshman gateway for biomedical sciences majors).

Experiment 2 (quasi-experimental, sections assigned): MIP 300 (junior-level, foundational microbiology; fall 2020 online due to pandemic).

## Results

### Practice behaviour changes

**Fig. 2.**
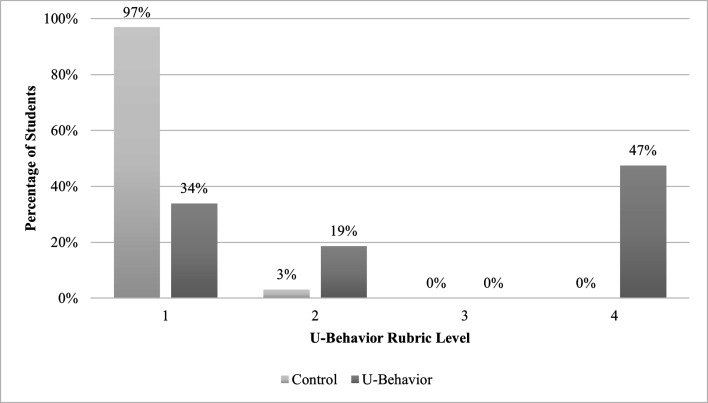
VMBS 100: distribution of UB-rubric scores. *Note*: Distribution of U-Behavior rubric levels in the control and U-Behavior conditions in VMBS 100. See also Table 4 for within-UB exam performance by rubric level.

**Fig. 3.**
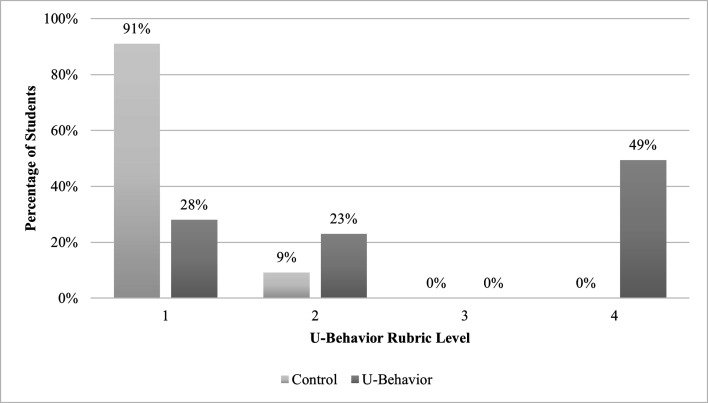
MIP 300: Distribution of U-Behavior rubric level. *Note*: Distribution of U-Behavior rubric levels in the control and U-Behavior conditions in MIP 300. See also Table 5 for within-UB exam performance by rubric level.

In both courses/experiments, U-Behavior significantly increased spacing and mixing (large effects; Tables 2 and 3) and a significant shift in rubric levels (*χ*²>56, *P*<0.001; Figs 2 and 3), with ~49–50% reaching Level 4 (Figs 2 and 3). The total RPA attempts did not differ between conditions in either experiment. This is an important finding as it would indicate that performance outcomes were based on the quality of the practice behaviour (spacing and mixing) and not simply quantity or number of attempts.

**Table 2.** Descriptive and inferential statistics of study behaviours by condition in VMBS 100

	Spacing		Mixing		Total attempts	
	Control	U-Behavior	Control	U-Behavior	Control	U-Behavior
Valid	65	59	65	59	65	59
Missing	0	0	0	0	0	0
Mean	2.82	56.50	16.67	71.33	38.62	43.58
sd	9.00	38.91	26.12	34.44	14.48	20.17
Welch’s t-test*	*t*(63.63)=10.35, *P*<0.001, *d*=1.90		*t*(107.72)=9.88, *P*<0.001, *d*=1.79		*t*(104.29)=1.56, *P*=0.12, *d*=0.28	

* The assumption of homogenous variances was violated for each test, which compared the control and U-Behavior conditions on each study behaviour measure.

**Table 3.** Descriptive and inferential statistics of study behaviours by condition in MIP 300

	Spacing		Mixing		Total attempts	
	Control	U-Behavior	Control	U-Behavior	Control	U-Behavior
Valid	77	87	77	87	77	87
Missing	0	0	0	0	0	0
Mean	10.3	56.5	24.5	72.1	34.5	30.7
sd	15.6	33.2	21.4	30.2	17.3	14.5
Welch’s t-test*	*t*(125.05)=11.77, *P*<0.001, *d*=1.82		*t*(154.48)=12.01, *P*<0.001, *d*=1.87		*t*(149.82)=−1.13, *P*=0.26, *d*=−0.12	

* The assumption of homogenous variances was violated for each test, which compared the control and U-Behavior conditions on each study behaviour measure.

**Table 4.** VMBS 100: within-UB exam performance by UB-rubric level

Outcome	Level 1 M (SD)	Level 2 M (SD)	Level 4 M (SD)	ANOVA	*η*²	L1 vs L4
Midterm	42.60 (4.07)	42.27 (3.77)	45.16 (2.11)	Welch’s *F*(2,21.74)=5.30, *P*=0.013	0.155	*d*=−0.80, *P*=0.016
Final exam	80.65 (7.36)	83.61 (6.26)	83.95 (6.76)	Welch’s *F*(2,27.60)=1.31, *P*=0.285	0.049	*d*=−0.48, *P*=0.121

**Table 5.** MIP 300: within-UB exam performance by UB-rubric level

Outcome	Level 1 M (SD)	Level 2 M (SD)	Level 4 M (SD)	ANOVA	*η*²	L1 vs L4
Exam 1	73.94 (11.13)	72.00 (15.50)	75.36 (15.65)	Welch’s *F*(2,45.82)=0.32, *P*=0.727	0.009	*d*=−0.10, *P*=0.667
Exam 2	71.56 (18.92)	76.97 (10.11)	79.28 (12.32)	Welch’s *F*(2,43.98)=1.63, *P*=0.207	0.053	
Exam 3	66.40 (19.69)	79.60 (13.00)	83.20 (13.26)	Welch’s *F*(2,42.05)=6.86, *P*=0.003	0.185	*d*=−1.10, *P*<0.001
	61.83 (20.15)	76.20 (8.92)	81.26 (9.24)	Welch’s *F*(2,40.40)=10.60, *P*<0.001	0.289	*d*=−1.48, *P*<0.001
ExamsFinalScore	68.43 (14.00)	76.20 (9.59)	78.77 (12.25)	Welch’s *F*(2,46.31)=4.54, *P*=0.016	0.117	*d*=−0.85, *P*=0.004

Prior academic performance (HS GPA/ACT/SAT in VMBS 100; university GPA in MIP 300) showed no association with UB-rubric level. To a certain extent, this negates the ‘rich-get-richer’ concern where only the good students engage and change their behaviour.

### Exam performance

No significant between-condition differences appeared in midterm/final exams (both courses).

### Within-condition analysis: practice quality and exam performance among U-Behavior students

To test whether the null between-condition exam differences reflected incomplete adoption of the U-Behavior practice patterns rather than absence of an effect of those patterns on learning, we conducted within-U-Behavior analyses comparing exam performance by UB-rubric level. Analyses use only confound-free outcomes (see Methods). UB-rubric level distributions in the U-Behavior group were 20/11/28 (Levels 1/2/4) in VMBS 100 and 24/20/43 in MIP 300; no students reached Level 3 in either course.

### VMBS 100 within-UB results (Table 4)

A meaningful within-condition gradient appeared on the Midterm [Welch’s *F*(2, 21.74)=5.30, *P*=0.013, *η*²=0.155; Level 4 vs Level 1 *d*=−0.80] but not on the Final Exam [Welch’s *F*(2, 27.60)=1.31, *P*=0.285; gradient in the predicted direction, *d*=−0.48].

### MIP 300 within-UB results (Table 5)

The within-condition gradient on the Final Exam was very large. Level 4 students scored nearly 20% points above Level 1 students (81.26 vs 61.83, *d*=−1.48). The four-exam composite (ExamsFinalScore) also showed a clear significant gradient (*d*=−0.85, *P*=0.004). A sensitivity analysis excluding the small number of zero-score ‘missed exam’ cases (1–2 students per outcome) did not change any conclusion and slightly strengthened the L1 vs L4 difference on Final Exam (*d*=−1.62, *P*<0.001).

#### Time-course pattern across MIP 300 exams

The effect of UB-rubric level on exam performance grew consistently across the four MIP 300 exams: *η*²=0.009 (Exam 1) → 0.053 (Exam 2) → 0.185 (Exam 3) → 0.289 (Final Exam). This time-course gradient is exactly what would be predicted if U-Behavior’s behavioural targets (spaced retrieval and interleaving) work by building durable knowledge over time. Early in the semester, before students have had enough time to accumulate spaced practice, there is no detectable effect of UB level on performance. The effect emerges mid-semester, becomes large by Exam 3 and reaches its maximum on the comprehensive Final Exam, providing direct empirical support for the durable-learning rationale that motivates the U-Behavior approach [1–4].

### Exploratory regressions (combining conditions)

**VMBS 100**: Spacing predicted midterm (*β*=0.29, medium effect) but not final scores; again, total attempts did not predict either.

**MIP 300**: Spacing predicted midterm (*β*=0.156), final (*β*=0.219), above university GPA and attempts (small-to-medium effects); attempts predicted final only (small effect).

Overall, higher spacing linked to better outcomes in these challenging courses, independent of study quantity or prior performance.

Robustness check: per-condition regressions. To address the question of whether the combined-condition regressions reported above reflect a genuine spacing→outcome relationship or a statistical artefact of pooling conditions with different baseline behaviours, we re-ran all regressions separately within each condition. In the control condition, practice behaviour was highly restricted, most students clustered at low spacing and mixing scores (Tables 2 and 3) which limits the regression’s statistical power to detect a behaviour→outcome relationship. In VMBS 100 control students (*n*=64), Spacing was non-significantly associated with both Midterm (*R*²=0.054, *P*=0.063) and Final Exam (*R*²=0.037, *P*=130); Mixing showed a significant association with Midterm (*R*² = 170, *P* < 0.001), but the effect was smaller at the Final Exam (*R*²=065, *P*=0.042). In MIP 300 control students (*n* =76), no spacing or mixing predictor reached significance for either outcome (all *R*²<0.05, all *P*>0.06), consistent with the restricted range of practice behaviour in that condition. In contrast, in the U-Behavior condition, where the intervention expanded the distribution of practice behaviours, spacing and mixing were consistently and substantially associated with exam performance. In VMBS 100 U-Behavior students (*n*=0.59), Spacing predicted Midterm performance (*R*²=0.131, *P*=0.005) and Mixing predicted both Midterm (*R*²=0.113, *P* = 0.009) and Final Exam (*R*²=0.082, *P*=0.028). In MIP 300 U-Behavior students (*n*=86), the associations were strongest: Spacing predicted Final Exam performance (*R*²=0.296, *P*<0.001) and four-exam composite score (*R*²=0.147, *P*<0.001), as did Mixing (Final Exam: *R*²=0.262, *P*<0.001; composite: *R*²=0.105, *P*=0.002). These per-condition results are coherent with the combined-condition regressions: the spacing→outcome relationship is detectable primarily in the condition where the intervention created meaningful variance in practice behaviour and the combined-condition analysis pools this variance to maximize statistical power. The pattern supports the interpretation that the regression results reflect a genuine association between practice quality and learning outcomes rather than a between-group confound.

## Discussion

U-Behavior effectively shifted study behaviours toward evidence-based practices in two courses essential for biomedical sciences majors: VMBS 100, a critical gateway course introducing biomedical concepts, and MIP 300, a content-intensive upper-division course covering microbial physiology, structure and molecular biology-foundational knowledge required for advanced microbiology, infectious disease and related STEM pathways. By significantly boosting spacing and interleaving of retrieval practice (large effects, ds >1.75) without increasing total study time, U-Behavior demonstrates that targeted coaching can improve study *quality*, aligning that behaviour with cognitive science principles that emphasize effortful, distributed retrieval for durable learning [1, 3, 4]. Importantly, this is done with little to no additional effort from the instructor.

The null between-condition differences in exam performance are best understood through a mediation lens combined with a content-difficulty interpretation. The within-U-Behavior analyses (Tables 4 and 5) directly test the incomplete-adoption explanation: students at Level 4 – those who fully adopted the U-Behavior practice patterns outperformed Level 1 students by ~20% points on the MIP 300 Final Exam (*d*=−1.48) and by smaller but still meaningful margins on VMBS 100’s Midterm (*d*=−0.80) and MIP 300’s four-exam composite (*d*=−0.85). Coupled with the time-course pattern across MIP 300 exams (*η*² growing from 0.009 on Exam 1 to 0.289 on the Final Exam), these results provide direct empirical support for the manuscript’s claim that practice quality matters for durable learning. The asymmetric pattern across courses, significant within-UB gradients on VMBS 100 Midterm but not Final Exam, and growing gradients across MIP 300 exams are consistent with a content-difficulty interpretation: VMBS 100 introductory content is light enough that even Level 1 students retain most of it through any study strategy, compressing the gradient by the Final Exam, whereas MIP 300 upper-division content (microbial structure, function, physiology and molecular biology) is taxing throughout, so practice quality matters increasingly as material accumulates. The argument structure for U-Behavior’s effect on learning is as follows: U-Behavior changes practice behaviour (causally identified through randomization in VMBS 100; large effects with d>1.75), and practice behaviour change is associated with substantially better performance on confound-free outcomes (within-UB analyses; large effect sizes). This is not a formal mediation test, but it is the strongest argument the design supports.

These findings carry direct implications for microbiology pedagogy. In demanding curricula like those for microbiology majors, where knowledge attrition is common due to volume and conceptual complexity, tools like U-Behavior can help students self-regulate more effectively. By leveraging existing LMS infrastructure for personalized visualizations (RPA graphs) and low-burden reflections, instructors can promote metacognitive awareness and combat common maladaptive habits (e.g. cramming) without overhauling course design. The method’s scalability – requiring minimal additional resources beyond quiz pools – makes it feasible for large-enrollment or online microbiology courses, including during disruptions like the pandemic (as in VMBS100 and MIP 300). Moreover, benefits appear independent of prior performance, supporting equity: lower-performing or first-generation students in STEM were quick to achieve effective practice patterns, potentially reducing achievement gaps in gateway and foundational microbiology classes.

Limitations include partial uptake (suggesting motivational or implementation barriers) and the quasi-experimental design in MIP 300, though randomization in VMBS 100 strengthens causal inferences for behaviour change. Future iterations could integrate behaviour change techniques (e.g. self-efficacy boosts via peer success stories or goal-setting prompts) to increase optimal adoption rates [11], potentially yielding condition-level performance differences. Additional research should test U-Behavior in other microbiology subfields (e.g. virology and immunology), explore long-term transfer to subsequent courses and assess impacts on student self-efficacy and attitudes toward science. Within-UB analyses use confound-free outcomes (Midterm and Final Exam in VMBS 100; Exam 1–Final Exam and ExamsFinalScore in MIP 300) rather than Final Score, because the U-Behavior practice grade contributes to Final Score and would inflate within-condition gradients mechanically. The within-UB evidence linking practice quality to outcomes is correlational rather than experimentally identified, and we did not test mediation formally; the unmeasured confounders that any correlational analysis is subject to should be borne in mind.

In summary, U-Behavior offers microbiology educators a practical, evidence-informed tool to foster spaced, interleaved retrieval practice in rigorous courses. By guiding students toward higher-quality studying, it has strong potential to enhance durable learning, support major progression and better prepare undergraduates for microbiology careers.

### Use of generative AI

During the preparation of this revision, the authors used Claude (Anthropic, 2026) as a writing and analysis assistant. Specific AI-assisted contributions were as follows: (1) organizing and triaging reviewer comments into a tracked revision plan; (2) summarizing and indexing referenced literature; (3) drafting prose for the new Measures subsection and the Within-condition analysis subsection added in this revision; (4) conducting initial exploratory within-condition statistical analyses, the results of which were subsequently re-run and verified by the authors in JASP (see Methods); and (5) preparing the tracked-changes revision document. All scientific insights, data interpretations and conclusions including the mediation framing, the content-difficulty interpretation of cross-course differences and the methodological decision to use confound-free outcomes for within-condition analyses were developed and approved by the authors. All AI-generated text was reviewed and verified by the authors, who take full responsibility for the manuscript’s content.

## Abbreviations

CSU: Colorado State University
GPA: Grade Point Average
HS: High School
LMS: learning management system
RPA: retrieval practice activity
STEM: Science, Technology, Engineering, and Mathematics
UB: U-Behavior
